# Enhancement of cryopreserved rooster semen and fertility potential after oral administration of Thai ginger (*Kaempferia parviflora*) extract in Thai native chickens

**DOI:** 10.5713/ab.24.0004

**Published:** 2024-04-01

**Authors:** Vibuntita Chankitisakul, Supakorn Authaida, Wuttigrai Boonkum, Sarunya Tuntiyasawasdikul

**Affiliations:** 1 Department of Animal Science, Faculty of Agricultural, Khon Kaen University, Khon Kaen, 40002, Thailand; 2 Network Center for Animal Breeding and Omics Research, Khon Kaen University, Khon Kaen, 40002, Thailand; 3 Center for Research and Development of Herbal Health Products, Faculty of Pharmaceutical Sciences, Khon Kaen University, Khon Kaen, 40002, Thailand

**Keywords:** Antioxidants, Frozen-thaw Semen, Indigenous Chicken, Lipid Peroxidation

## Abstract

**Objective:**

Semen cryopreservation is an effective method of preserving genetic material, particularly in native chicken breeds facing a substantial decline. In this study, we evaluated the quality of frozen/thawed rooster semen treated with different concentrations of oral administrations of black ginger (*Kaempferia parviflora*: KP) extract and determined its fertility.

**Methods:**

Thirty-two Thai native roosters (Pradu Hang Dum, 42 weeks old) were used in this study. The treatments were classified into four groups according to the concentration of KP extract administered to the roosters: 0, 100, 150, and 200 mg/kg body weight. The quality of fresh semen was analyzed before cryopreservation. Post-thaw sperm quality and fertility potential were determined. Also, lipid peroxidation was determined.

**Results:**

The results showed that sperm concentration and movement increased in roosters treated with 200 mg/kg of KP extract (p<0.05). The malondialdehyde (MDA) in the roosters receiving 200 mg/kg KP extract was lower than that in the other but had an insignificant difference within the KP treatment groups (p>0.05). The highest MDA levels were observed in the control group (p<0.05). The percentage of motile sperm (total motility and progressive motility) after semen thawing was higher in roosters that received 150 and 200 mg/kg KP extract than in those that received 100 mg/kg KP extract and the control (p<0.05). MDA levels decreased significantly in roosters that received 150 and 200 mg/kg KP extract than in those that received 100 mg/kg KP extract and the control (p<0.05). Fertility and hatchability were greater in the KP150 and KP200 groups than in the KP100 and control groups (p<0.05).

**Conclusion:**

The optimal amount of KP extract influencing initial sperm quality was determined to be 200 mg/kg. However, 150 mg/kg was the optimal low dosage of KP extract administration that maintained sperm quality and fertility following semen cryopreservation.

## INTRODUCTION

Native chickens play a crucial role for the majority of smallholder farmers in low- and middle-income countries, including Thailand. Various Thai native breeds are known to be valued for their taste and flavor [1]. However, the number of farmers raising native chickens is decreasing, probably because of their comparatively lower growth performance, which is less competitive than that of commercial breeds. Additionally, the substantial increase in feedstuff costs contributes to this trend. Therefore, the conservation of the genetic resources of these species is imperative; otherwise, these valuable chickens would become endangered or extinct. As a component of reproductive conservation strategies, semen cryopreservation, identified as the only option for preserving gamete cells [2], emerges as a preferable procedure. However, the ability to freeze and thaw sperm depends on several factors, including initial semen quality and cryotolerance during semen cryopreservation, which have deleterious effects on sperm characteristics owing to sperm plasma membrane damage.

Thailand has a largely tropical climate with a high ambient temperature and humidity throughout the year. Therefore, Thai native chickens raised in an open-house environment are affected by heat stress [3,4]. Increased body temperature increases metabolism and oxygen consumption to sustain metabolism, leading to tissue oxidative stress [5]. This situation occurs in all organs of the body, including the testes. Exposure to heat stress induces abnormalities in spermatogenesis and reduces testosterone production [6]. Furthermore, heat stress-induced low sperm quality is associated with increased production of reactive oxygen species (ROS) production and subsequent lipid peroxidation [7]. The quality of Thai native rooster sperm significantly decreases mainly during the summer and rainy seasons [8]. Environmental cooling, such as an evaporative cooling system, may be an option; however, it is not practical for small farmers in rural areas.

In addition, sperm cells exhibit resistance towards membrane phospholipids in the cryopreservation process [9]. Thai native rooster sperm contains high levels of polyunsaturated fatty acids (PUFAs) in their phospholipid composition of the plasma membrane, mainly arachidonic acid (C20:4n-6) and docosahexaenoic acid (C22:6n-3) [10], making sperm cells very susceptible to excess ROS owing to lipid peroxidation [11]. Damage to the sperm plasma membrane results in a decrease in fertilizing ability. Therefore, antioxidants that can scavenge ROS are required to prevent sperm membrane damage.

*Kaempferia parviflora* (KP) or “Thai ginger,” or in Thai “Krachaidum,” is a traditional Thai herb containing several bioactive components mainly containing methoxy flavones, especially 3,5,7,3′,4′-pentamethoxyflavone (PMF), 5,7-dimethoxyflavone (DMF), and 5,7,4′-trimethoxyflavone (TMF) [12]. KP is widely used in traditional Thai medicine for several medical purposes, including its recognized anti-inflammatory, antimicrobial, and anticancer properties in human [13–15]. In the context of sexual enhancement, consumers believe that KP increases male sexual activity and promotes reproductive functions [16]. The KP increases the semen volume, sperm count, sperm motility, and viability of rabbit semen [17]. The KP also enhances spermatogenesis in rats [18,19]. This might be the result of the vasodilatory effect of flavonoids in KP [20–22], subsequently stimulating testosterone production, which, in turn, influences spermatogenesis [23,24]. Furthermore, the antioxidant components and activities of KP have recently been documented [25]. The finding could be beneficial in utilizing KP as an antioxidant compound.

One solution to diminish the effects of environmental stress on rooster fertility, together with protecting against sperm membrane damage during cryopreservation, might be simultaneously accomplished by feeding the roosters a substance that would potentially improve sperm production and reduce ROS accumulation. As mentioned previously, KP appear to have the desired properties. Although KP has been widely used, no study has been conducted on the effects of KP supplementation on semen cryopreservation in any species. We hypothesized that supplementation of roosters with KP would positively affect sperm production and semen cryopreservation.

## MATERIALS AND METHODS

### Materials

The KP extract was obtained from the Center for Research and Development of Herbal Health Products at Khon Kaen University. According to the laboratory procedure, KP was dried in a hot air oven at 60°C for 48 h and ground into powder. The powdered KP was macerated with 95% ethanol in a stainless-steel tank for 3 days and evaporated through rotary evaporation. The KP extract used in the current study was previously analyzed via high-performance liquid chromatography using the methods described by Tuntiyasawasdikul et al [26]. The components of the KP extract have been reported in our previous study conducted by Authaida et al [27]. In that study, the components of KP extract were reported to be 25.19 mg/g PMF, 22.94 mg/g DMF, and 42.54 mg/g TMF. The KP extract was stored at −20°C until being used. Before oral supplementation with KP, the KP extract was prepared in a mixture of propylene glycol (28%), polyethylene glycol 400 (35%), ethanol (2%), and deionized water (adjusted to 100%) at a concentration of 150 mg/mL.

Unless otherwise stated, all chemicals used in this study were purchased from Sigma-Aldrich Chemical Co. (St. Louis, MO, USA).

### Animals and management

The Institutional Animal Care and Use Committee approved the experimental protocol based on the Ethics of Animal Experimentation of the National Research Council of Thailand (record no. IACUC-KKU-29/65; Reference no. 660201.2.11/ 197 (33)). Thirty-two Thai native roosters (Pradu Hang Dum, 42 weeks old) were used in this study. They were housed in individual cages (60×45×45 cm) in an open-house system. Before initiating the experiment, 130 g of a commercial diet without KP extract supplementation was administered per day, and water was provided *ad libitum*.

Forty-eight Thai native hens (Pradu Hang Dum) at 46 weeks of age with egg production >60% were used for the fertility test. The hens were housed individually, fed approximately 110 g of a commercial diet daily, and provided with water *ad libitum*.

### Experimental design

The roosters were randomly divided into four groups (eight roosters per group) according to the concentration of KP extract (0, 100, 150, and 200 mg/kg of body weight). A 10 mL syringe was used to orally administer the KP extract suspension to the KP treatment groups. The roosters in each treatment group received oral administration of KP extract for a minimum of 14 days before semen collection, and the supplementation continued until the conclusion of the experiment. The control group received the solvent without KP extract. The fresh semen volume, sperm concentration, mass movement score, sperm viability and lipid peroxidation were evaluated. The semen was diluted with a semen extender and subsequently subjected to the freezing process. The total motility (MOT), progressive motility (PMOT), viability, and lipid peroxidation levels were evaluated after thawing at 5°C. The experiment on semen cryopreservation and semen evaluation was conducted with six replicates. Additionally, the fertilizing ability of the frozen–thawed semen was tested with 48 Thai native hens. They were randomly assigned to one of four treatment groups (twelve hens/group) first and swapped to other treatment groups at an interval of a week; therefore, all hens were inseminated with all treatment groups. The fertility test was replicated four times.

### Semen collection

The dorsoabdominal massage technique was used twice weekly for semen collection. Individual rooster semen samples were collected in 1.5 mL microtubes containing 0.1 mL of Schramm extender [28] and maintained at a temperature of 22°C to 25°C during transportation, completed within 15 min, to the laboratory for further analyses. Immediately after transport, each semen sample was individually evaluated for volume, sperm concentration, and sperm mass movement. Semen with the criteria of semen volume, at or above 0.3 mL; sperm concentration, ≥3×10^9^ spz/mL; and motility score, ≥4, were pooled to eliminate the individual effects for further semen cryopreservation.

### Semen cryopreservation

Cryopreservation was performed by diluting the pooled semen with a Schramm extender at 1:3 (v:v) and cooling it to 5°C for 1 h. Subsequently, N, N-dimethylformamide was added to a semen extender at a final concentration of 6% (v/v) before loading into 0.5 mL plastic straws and sealing with polyvinyl powder, followed by equilibration for 15 min. The straws were then placed horizontally on a rack 11 cm and 3 cm above the surface of liquid nitrogen in a Styrofoam box for 12 and 5 min, respectively. Thawing was performed at 5°C for 5 min in cool water. Freezing and thawing were conducted as described in our previous study [29].

### Assessment of sperm motility

The motility of fresh semen was assessed based on the intensity of waves formed by sperm movement (mass movement). A drop of 10 μL semen was placed on a slide without a coverslip, examined under a compound microscope (100×), and scored on a scale of 0 to 5 (0 = no sperm movement; 5 = very rapid waves and whirlwinds visible, with more than 90% of sperm showing a forward movement).

A computer-assisted sperm analysis (CASA) system (version 10 HIM-IVOS; Hamilton Thorne Biosciences, Beverly, MA, USA) was used to assess MOT and PMOT of frozen semen. The samples were evaluated in a chamber slide, maintained at 25°C, with 5 μL per sample. Three fields were recorded using a 10× phase-contrast objective (Olympus, Tokyo, Japan) in conjunction with a digital camera (DP 71/25; Olympus, Japan). CASA settings were configured at 30 frames per second (60 Hz). Sperm was defined as nonmotile when the average path velocity was less than 5 μm/s, while sperm was considered progressively motile when the average path velocity was greater than 20 μm/s, along with a straightness index of 80%.

### Assessment of sperm viability

Sperm viability of fresh semen was assessed by staining with eosin/nigrosine. A 5 μL semen sample was mixed with 10 μL of staining and smeared on a slide. A total of 300 sperms were counted under a light microscope (CH30; Olympus, Japan) and classified as dead (pink) or live (colorless).

The sperm viability of frozen semen was determined by staining with SYBR-14 and propidium iodide (PI) (LIVE/DEAD Sperm Viability Kit; Invitrogen TM, Thermo Fisher Scientific, Waltham, MA, USA). The semen sample was first incubated with 5 μL of SYBR-14 solution for 10 min and then stained with 5 μL PI for 5 min. Following this, sperm in each sample was fixed by adding 10 μL of 10% formaldehyde and analyzed within an hour. At least 300 sperms were counted under an IX71 fluorescence microscope (Olympus, Japan) and classified as dead (red sperm) or live (green sperm).

### Assessment of malondialdehyde

The extent of lipid peroxidation in the semen samples was determined by measuring the malondialdehyde (MDA) concentration. Semen samples (250 μL) from each treatment group were adjusted to a concentration of 250×10^6^ spz/mL. The samples were combined with 0.25 mL ferrous sulfate (0.2 mM, 12354; Sigma, USA) and 0.25 mL ascorbic acid (1 mM, A5960; Sigma, USA), followed by incubation at 37°C for 60 min. Next, 1 mL trichloroacetic acid (15%, T6399; Sigma, USA) and 1 mL thiobarbituric acid (0.375%, T550; Sigma, USA) were added, and the mixture was boiled in water for 10 min. The samples were then cooled to 4°C to stop the reaction. Finally, the samples were centrifuged at 4,000×g for 10 min at 4°C. Supernatants (2 mL) were analyzed using a UV-visible spectrophotometer (Analytik Jena Model Specord 250 plus) at 532 nm.

### Fertility

The fertility test was conducted by artificially inseminating hens (twelve hens/group) with 0.4 mL of frozen-thawed semen once a week. Insemination was performed between 15.00 and 17.00 h. Eggs collected from days 2 and 8 after insemination were incubated in an incubator for 21 days. Fertility was determined by candling eggs on day 7 of incubation. The hatchability of fertile eggs was determined on day 21 of incubation. Fertility was calculated as the percentage of fertile eggs relative to the total eggs. The hatchability of fertile eggs was calculated as a percentage of total eggs.

### Statistical analysis

Data on semen quality was conducted using an analysis of variance (ANOVA) with a completely randomized design and six replicates. ANOVA analyzed data on fertility ability as a cross-over design and four replicates. The study factors included treatment groups, the insemination period, and chicken hens, which were statistically tested. Treatment means were compared using Tukey’s range test to determine significant differences at p<0.05 for each parameter. Data were recorded as percentages and transformed using the arcsine method prior to statistical analysis. The results are presented as the mean±standard error of the mean.

## RESULTS

### Fresh semen analysis

The quality of fresh semen from roosters that received oral supplementation with KP extract is presented in Table 1. Sperm concentration and movement (determined as mass movement) increased in roosters treated with 200 mg/kg KP extract (p<0.05). Simultaneously, those of the other KP groups and the control group did not differ significantly (p>0.05). Sperm viability was significantly higher in the roosters that received 200 mg/kg KP extract but did not differ from those that received 150 mg/kg KP extract (p>0.05). Semen volume did not differ between groups (p>0.05). MDA levels in roosters treated with 200 mg/kg KP extract were lower than those in the other groups. However, this difference was not significant in the KP treatment groups (p>0.05). The highest MDA level was observed in the control group compared to the other groups supplemented with KP extract. However, this difference was found only in roosters treated with 200 mg/kg KP extract (p<0.05).

### Frozen semen analysis

The quality of frozen semen from roosters, with and without oral supplementation of KP, is presented in Table 2. The percentage of motile sperm (MOT and PMOT) after semen thawing was higher in the roosters that received 150 and 200 mg/kg KP extract compared to those that received 100 mg/kg KP extract and the control group (p<0.05). Correspondingly, MDA levels decreased significantly in roosters that received 150 and 200 mg/kg KP extract compared with those that received 100 mg/kg KP extract and the control group (p<0.05). However, the highest sperm viability was observed in roosters treated with 200 mg/kg KP extract.

### Fertility ability

ANOVA with a cross-over design revealed significant differences between the treatment, but not between the insemination period as well as between animal effects. Table 3 shows the fertility and hatchability percentages after artificially inseminating hens with frozen semen. Fertility and hatchability were higher in the KP150 and KP200 groups than in the KP100 and control groups (p<0.05).

## DISCUSSION

Sperm cryopreservation is crucial to preserve the genetic resources of native chickens; however, damage from cryopreservation reduces sperm quality and fertility. In addition, heat stress from environmental stress can significantly reduce the initial sperm quality. To the best of our knowledge, this is the first study to demonstrate the beneficial effects of orally administered KP extract on rooster sperm cryopreservation. The results of this study indicated that sperm density and movement increased when roosters received 200 mg/kg of KP extract. Similar results have been reported previously [27], thus confirming the possibility of using KP extract at 200 mg/kg administration for providing the most effective of most ejaculated sperm parameters. However, the sperm movement, MDA concentrations, and fertility ability of frozen semen from roosters that received 150 and 200 mg/kg KP extract did not differ, indicating that 150 mg/kg was the optimal low dosage of KP extract administration for the roosters which acts as an antioxidant protecting the sperm from lipid peroxidation during cryopreservation processing. Interestingly, the lowest effective dose of KP extract supplementation on frozen semen in this study (150 mg/kg) was lower than that in cooled semen in a previous study (200 mg/kg) [27]. The difference might be a result of the independent severity of oxidative stress between semen preservation procedures.

Heat stress from environmental increases spermatogenic abnormalities, reduces testosterone production, and increases lipid peroxidation [6,7]. The findings of the present study showed a positive effect of KP on sperm quality, as evidenced by increased sperm density, movement, and viability in the group that received 200 mg/kg KP extract compared to the other groups. This finding is consistent with that of our previous report [27]. Sperm production is improved by the vasodilatory effect of KP, which relaxes the smooth muscle and increases blow flow to the testis [30]. This results in a higher sperm concentration and increased sperm motility. Decreased lipid peroxidation, as evidenced by lower MDA concentrations, may also result from another function of KP, which contains antioxidant components (phenolics, flavonoids, and anthocyanins) and antioxidant activities (2,2-diphenyl-1-picrylhydrazyl and ferric ion reducing antioxidant power) [25]. Flavonoids can protect against free radical damage via pathways such as direct scavenging of ROS, activation of antioxidant enzymes, and inhibition of oxidases [31]. In avian semen, the high PUFAs in the sperm plasma membrane increase susceptibility to lipid peroxidation by ROS. MDA, an index of lipid peroxidation and a marker of oxidative stress, increases during in vivo sperm storage [32]. MDA levels in the seminal plasma have been reported to be negatively correlated with sperm motility [10]. Thus, the increased antioxidant components and enzyme activities could be a protective response against lipid peroxidation, subsequently decreasing the MDA content of fresh semen, as presented in Table 1.

KP functions exhibit antioxidant properties that extend beyond fresh semen, positively impacting semen freezing by protecting sperm plasma membrane from oxidative stress-induced damage during cryopreservation. After freezing and thawing, sperm undergo an increase in ROS levels, indicating that oxidative stress occurs during this process [33]. In contrast, the levels of natural antioxidants that protect sperm from oxidative damage are low [34]. These characteristics make the sperm susceptible to lipid peroxidation, resulting in sperm damage and reduced fertility. Supplementation with antioxidants, such as vitamin E and selenium, in the diet is one of the alternative choices to increase the activity of antioxidant enzymes and subsequently reduce ROS production [35–37]. Compared with the control, the cryopreserved semen of the roosters that received KP extract showed increased sperm motility and viability (Table 2). KP extract levels were significantly correlated with sperm quality. The KP extract at 100 mg/mL did not affect the MDA concentration. In contrast, 150 and 200 mg/mL reduced MDA production in frozen semen, which helped maintain sperm quality during cryopreservation. Meanwhile, the fertility ability between 150 and 200 mg/ml doses did not significantly differ (Table 3). Therefore, it might be inferred that 150 mg/kg was the lowest dose that is effective for the results; in other words, 150 mg/kg was the optimal low dosage of KP extract administration to the roosters for maintaining sperm quality and fertility after semen cryopreservation.

Interestingly, the lowest effective dose of KP extract supplementation on frozen semen in this study (150 mg/kg) was lower than that in cooled semen in a previous study (200 mg/kg) [27]. This raises questions about whether the oxidative stress that occurs during cooling or freezing during storage is more pronounced. Semen cryopreservation involves several steps: dilution, cooling, cryoprotectant addition, freezing, and thawing. Thus, sperm undergoes cold shock, ice crystal formation, and osmotic stress during freezing, resulting in more deleterious effects in terms of lower sperm quality and reduced fertility potential than liquid storage [38]. Concurrently, MDA concentrations in frozen semen in the present study were higher than those in liquid semen in our previous study [27]. The current knowledge is limited; additionally, no prior studies have compared severe oxidative stress between cooled and cryopreserved semen storage. Questions regarding the differential impact of antioxidant supplements, determining which method results in increased oxidative stress, and elucidating the functioning of antioxidant enzymes remain unanswered. Further studies are warranted to address these questions.

## CONCLUSION

Our findings indicate that the optimal amount of KP extract influencing initial sperm quality was determined to be 200 mg/kg. However, 150 mg/kg was the optimal low dosage of KP extract administration that maintained sperm quality and fertility following semen cryopreservation.

## Figures and Tables

**Table 1:** Effects of different concentrations of KP extract oral supplementation on fresh semen quality in native rooster

Treatment^1)^	Concentration (×10^9^/mL)	Volume (mL)	Mass movement (Score 1 to 5)	Viability (%)	MDA (250×10^6^ spz/nmol/mL)
Control	3.3±0.45^b^	0.40±0.02	4.53±0.06^b^	93.41±0.48^c^	1.28±0.11^b^
KP100	3.3±0.41^b^	0.50±0.03	4.52±0.05^b^	93.96±0.51^bc^	1.19±0.09^ab^
KP150	3.5±0.45^b^	0.49±0.05	4.56±0.06^b^	95.28±0.53^ab^	1.16±0.08^ab^
KP200	4.1±0.46^a^	0.51±0.03	4.71±0.03^a^	96.65±0.53^a^	1.08±0.12^a^

KP, *Kaempferia parviflora*; MDA, malondialdehyde.

1) KP100, roosters administered 100 mg/kg KP extract; KP150, roosters administered 150 mg/kg KP extract; KP200, roosters administered 200 mg/kg KP extract.

a–c Means in a column with different letters differ (p<0.05; Tukey’s range test).

**Table 2:** Effects of different concentrations of KP extract oral supplementation on frozen semen quality in native rooster

Treatment^1)^	MOT (%)	PMOT (%)	Viability (%)	MDA (250×10^6^ spz/nmol/mL)
Control	48.01±0.38^b^	26.65±0.45^b^	53.81±0.43^c^	2.91±0.06^b^
KP100	46.87±0.43^b^	26.68±0.34^b^	53.64±0.49^c^	2.88±0.06^b^
KP150	54.54±0.54^a^	31.72±0.41^a^	59.07±0.61^b^	2.60±0.03^a^
KP200	55.85±0.41^a^	33.62±0.39^a^	62.21±0.51^a^	2.55±0.05^a^

KP, *Kaempferia parviflora*; MOT, total motility; PMOT, progressive motility; MDA, malondialdehyde.

1) KP100, roosters administered 100 mg/kg KP extract; KP150, roosters administered 150 mg/kg KP extract; KP200, roosters administered 200 mg/kg KP extract.

a–c Means in a column with different letters differ (p<0.05; Tukey’s range test).

**Table 3:** Effects of different concentrations of KP extract oral supplementation on fertility and hatchability in native hens

Treatment^1)^	Fertility (%)	Hatchability (%)
Control	60.18±0.73^b^	53.00±0.42^b^
KP100	61.27±0.64^b^	52.91±0.65^b^
KP150	63.86±0.53^a^	55.92±0.48^a^
KP200	65.18±0.61^a^	56.04±0.50^a^

KP, *Kaempferia parviflora*.

1) KP100, roosters administered 100 mg/kg KP extract; KP150, roosters administered 150 mg/kg KP extract; KP200, roosters administered 200 mg/kg KP extract.

a,b Means in a column with different letter differ (p<0.05; Tukey’s range test).
